# AGING WITH INCARCERATION HISTORIES: AN INTERSECTIONAL EXAMINATION OF INCARCERATION AND HEALTH AMONG OLDER ADULTS

**DOI:** 10.1093/geroni/igae098.1192

**Published:** 2024-12-31

**Authors:** Kenzie Latham-Mintus, Monica Deck, Elizabeth Nelson

**Affiliations:** Indiana University Indianapolis, Indianapolis, Indiana, United States; Indiana University Indianapolis, Indianapolis, Indiana, United States; Indiana University Indianapolis, Indianapolis, Indiana, United States

## Abstract

**Objectives:**

Experiences with incarceration are linked to poor mental and physical health across the life course. The pur- pose of this research is to examine whether incarceration histories are associated with worse physical and mental health among older adults. We apply an intersectionality framework and consider how the intersection of sexism and racism leads to unequal health outcomes following incarceration among women and people of color.

**Methods:**

We employ 2 measures of health (i.e., number of depressive symptoms and physical limitations) to broadly cap- ture mental and physical health. Using data from Waves 11 and 12 of the Health and Retirement Study, we estimated a series of general linear models to analyze differences in health by incarceration history, gender/sex, and race/ethnicity.

**Results:**

Findings suggest that experiences with incarceration are associated with a greater number of physical limitations and more depressive symptoms among older men and women, net of sociodemographic characteristics, early-life condi- tions, and lifetime stressful events. Formerly incarcerated women, particularly women of color, had more physical limita- tions and depressive symptoms relative to other groups.

**Discussion:**

These findings suggest that incarceration histories have far-reaching health implications. Older women of color with incarceration histories experience markedly high levels of physical limitations and depressive symptoms in later life.

